# Elective Lumbar Spine Surgery Leads to the Development of Chronic Pancreatitis and Pancreatic Pseudocyst: A Case Report and Literature Review

**DOI:** 10.7759/cureus.70272

**Published:** 2024-09-26

**Authors:** John G Skedros, Jessie A Montgomery, John T Cronin, Robert C Moesinger, Sujata D Kaushal, Paul J Johnson

**Affiliations:** 1 Shoulder and Elbow Surgery, Utah Orthopaedic Specialists, Salt Lake City, USA; 2 Orthopaedics, University of Utah, Salt Lake City, USA; 3 General Surgery, McKay-Dee Hospital, Ogden, USA; 4 Hospital Medicine, Mckay-Dee Hospital, Ogden, USA; 5 Orthopaedics, Mountain Orthopaedics, Bountiful, USA

**Keywords:** chronic pancreatitis, cyst-jejunostomy, lateral lumbar interbody fusions, pancreatic psuedocyst, posterior spinal fusion and instrumentation

## Abstract

The occurrence of pancreatitis shortly after elective lumbar spine surgery in an adult is rare. We report a case of a 63-year-old female who developed, for the first time, acute pancreatitis within three days of elective lumbar (L) spine surgery that was performed for degenerative disk disease without significant deformity (i.e., no scoliosis or spondylolisthesis). The surgery was conducted using a lateral transpsoas approach and included interbody fusions at L3-L4 and L4-L5 levels and posterior instrumentation with pedicle screws and rods. Ten years prior, she had a cholecystectomy, and she was not diabetic or obese. She began experiencing significant nausea and malaise two days after that lumbar spine surgery, requiring hospitalization on the third postoperative day. Her pancreatitis became chronic, and a large pancreatic pseudocyst developed and persisted despite using an external drainage catheter for 52 days. At 126 days after the spinal surgery, an open Roux-en-Y pancreatic cystojejunostomy was performed to internally drain the cyst, which had enlarged to 19 cm. Significant pre-surgical risk factors for this first-time case of pancreatitis were not identified. The spine surgeon denied iatrogenic causes such as instrument plunging or complications associated with the use of a "lateral access retraction system," and surgical blood loss was only 50 ml during the elective lumbar spine surgery. However, during the lumbar spine surgery, hypotension occurred for 20 minutes (mean arterial pressure: 63-73 mmHg), which was associated with transient acute kidney injury. This might have contributed to the development of her pancreatitis because the pancreas is more sensitive to ischemia than the kidney. During the initial week after the onset of pancreatitis, her symptoms were mainly believed to be due to an acute postoperative infection. However, there was no growth in cultures from aspirations of the pleural effusion, retroperitoneal effusion, and deeper incision area. Despite extensive workup, the cause of the patient's pancreatitis was not determined. We report this case not only because of its rarity but also to help surgeons and other healthcare providers in the workup and management of similar situations.

## Introduction

We report the case of a 63-year-old female who developed pancreatitis and a large pancreatic pseudocyst after elective lumbar (L) spine surgery for degenerative disc disease that was not associated with deformity (e.g., no scoliosis or significant spondylolisthesis). She had no clear risk factors for this index episode of acute postoperative pancreatitis and had an uncomplicated cholecystectomy nearly 10 years before. Because her pancreatic pseudocyst did not resolve with external drainage (52 days duration), an open Roux-en-Y pancreatic cystojejunostomy was performed 126 days after her lumbar spine surgery. This makes her case extraordinarily rare. In order to more fully understand the rarity, complexity, and treatment of her postoperative complication, it is useful to consider these issues in the context of the more common occurrence of postoperative pancreatitis in pediatric patients who had surgery to correct scoliosis deformities.

Pancreatitis after spine surgery: adult versus pediatric distinction

The occurrence of pancreatitis shortly after lumbar or thoracolumbar spine surgery is a well-documented complication in adolescents who have surgery to correct scoliosis deformities, with the reported incidence ranging from approximately 7-55% [1-5]. This broad range reflects differences in how the diagnosis of pancreatitis was made between these studies. Children in these cases fare well, with a vast majority of reported cases experiencing full recovery within weeks. In contrast to the high incidence of pancreatitis in adolescents after scoliosis surgeries, there are few reports of adults experiencing pancreatitis following thoracolumbar, lumbar, or lumbosacral surgery for any indication [6]. For example, Kobayashi et al. [7] reported on amylase levels in 262 adult patients (average age: 53 years) who had posterior spine surgery in the prone position for non-scoliosis conditions. Six of these patients had high levels of amylase (2.3% incidence), with one of these having acute pancreatitis (high levels of amylase and lipase). Our literature search (see below) revealed that all reported cases of pancreatitis following spinal surgery at various spinal levels in adults resulted in acute pancreatitis only, with just one case of recurrent pancreatitis in a 28-year-old female at six, 16, and 32 months after surgery (combined anterior and posterior instrumentation for neuropathic/polioscoliosis) [8]. That patient was fully resolved at the last reported follow-up visit.

Pancreatic pseudocysts

We identified two cases of a pancreatic pseudocyst that formed after spine surgery in adolescents: (1) a 14-year-old female who began experiencing progressive nonspecific abdominal symptoms soon after corrective spinal surgery for scoliosis and was diagnosed with acute pancreatitis (described as a “pancreatic fracture”) and pseudocyst formation [9], and (2) a 12-year-old male who had surgery to correct spinal deformity associated with hypotonic-pattern cerebral palsy [4]. Pancreatic pseudocyst is a serious diagnosis because of the risk for rupture or infection and can be difficult to treat [10]. In a review of English-language articles dealing with clinical characteristics and outcomes of acute pancreatitis following spine surgery published through May 2020 [6], and in our review of all articles in all languages up through May 2024, no cases were found of an adult with formation of a pancreatic pseudocyst in this postoperative context.

## Case presentation

**Figure 1:**
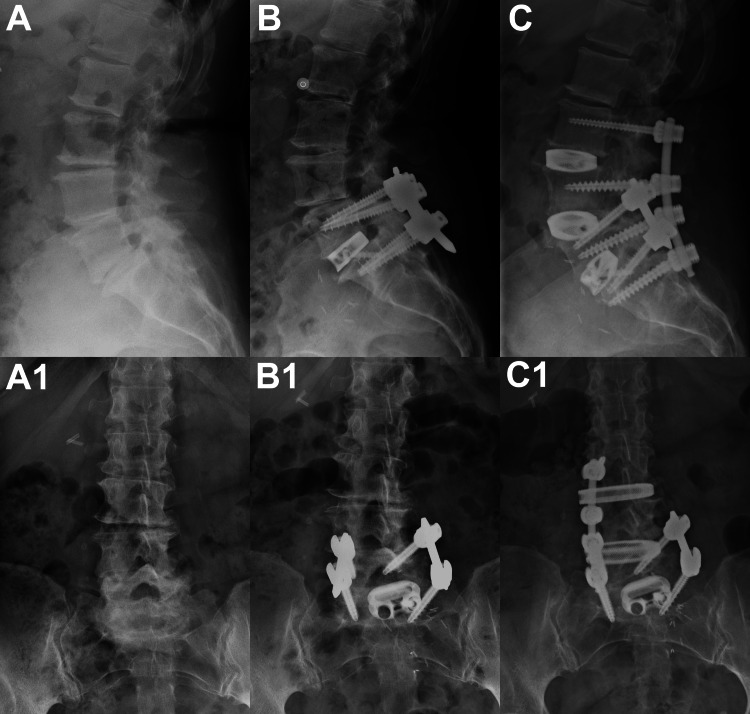
Radiographs of our patient's lumbar spine surgeries. Lateral (A) and anterior-posterior (A1) radiographs taken a few weeks before our patient’s first lumbar spine surgery. Lateral (B) and anterior-posterior (B1) radiographs after the second lumbar spine surgery, which included L5-S1 interbody fusion with posterior instrumentation using pedicle screws and left and right rods. Lateral (C) and anterior-posterior (C1) radiographs of the third (and final) lumbar spine surgery (November 2022), which included L3-L4 and L4-L5 interbody fusions with posterior instrumentation using pedicle screws and rods (long on the left and short on the right).

A 63-year-old female presented to her spine surgeon with a past medical history of two lumbar spine surgeries: (1) L4-L5 hemilaminectomy with left-sided microdiscectomy (first lumbar spine surgery) and (2) L5-S1 anterior discectomy and interbody fusion with posterior instrumentation (second lumbar spine surgery). Both of these surgeries were elective and performed for degenerative disc disease without significant deformity (e.g., no scoliosis, spondylolisthesis, etc.). These two prior surgeries had no significant complications and were performed on May 24, 2021, and January 14, 2022. Her second lumbar spinal surgery (the L5-S1 spinal fusion) involved the insertion of pedicle screws in L5-S1 with left and right rods that spanned this disc space (Figure 1).

The third, and final, lumbar surgery is described below and was performed on November 14, 2022, for back pain with sciatica. All three surgeries were done by the same fellowship-trained orthopedic spine surgeon (PJJ). None of the other authors of this case report were present at any of these surgeries, and none were colleagues or associates of the spine surgeon.

At the time of her third surgery, our patient was 155 cm tall and weighed 59.2 kg (BMI = 24.6); she had approximately the same BMI for her entire adult life. She did not smoke, was not diabetic, did not have a history of renal or pancreatic problems, and did not have a history of alcohol use disorder or binge drinking (though she drank two to three alcoholic beverages per week). This history is important because up to 25% of cases of acute pancreatitis and 70% of cases of chronic pancreatitis are secondary to chronic heavy alcohol consumption [11,12]. The Centers for Disease Control and Prevention, USA, defines heavy drinking as 15 or more drinks per week for men and eight or more drinks per week for women, though chronic pancreatitis requires this level of drinking to occur for multiple years [13]. Her medications included amlodipine for hypertension, desvenlafaxine for depression, estradiol for menopause symptoms, gabapentin for spine-related neuropathic pain, and zolpidem for insomnia. She had a prior uncomplicated cholecystectomy in January 2013 and prior to that date had an uncomplicated abdominal hysterectomy.

At the time of her third lumbar spine surgery in November 2022, she was COVID-19 negative and was not suspected of having any other viral illness. Furthermore, she was not suspected of having residual gallstones, was not hypercalcemic or hypertriglyceridemic, and did not have any conditions that usually cause hypertriglyceridemia. She also did not have other known risk factors for developing acute postoperative pancreatitis, including no history of endoscopic retrograde cholangiopancreatography (ERCP) and none of the following: recent trauma, cardiac bypass surgery, scorpion bites, exposure to large concentrations of organophosphates, kidney abnormalities, or any known anatomic variations of her hepatobiliary system [14].

Third spine surgery (two additional lateral lumbar interbody fusions) 

**Figure 2:**
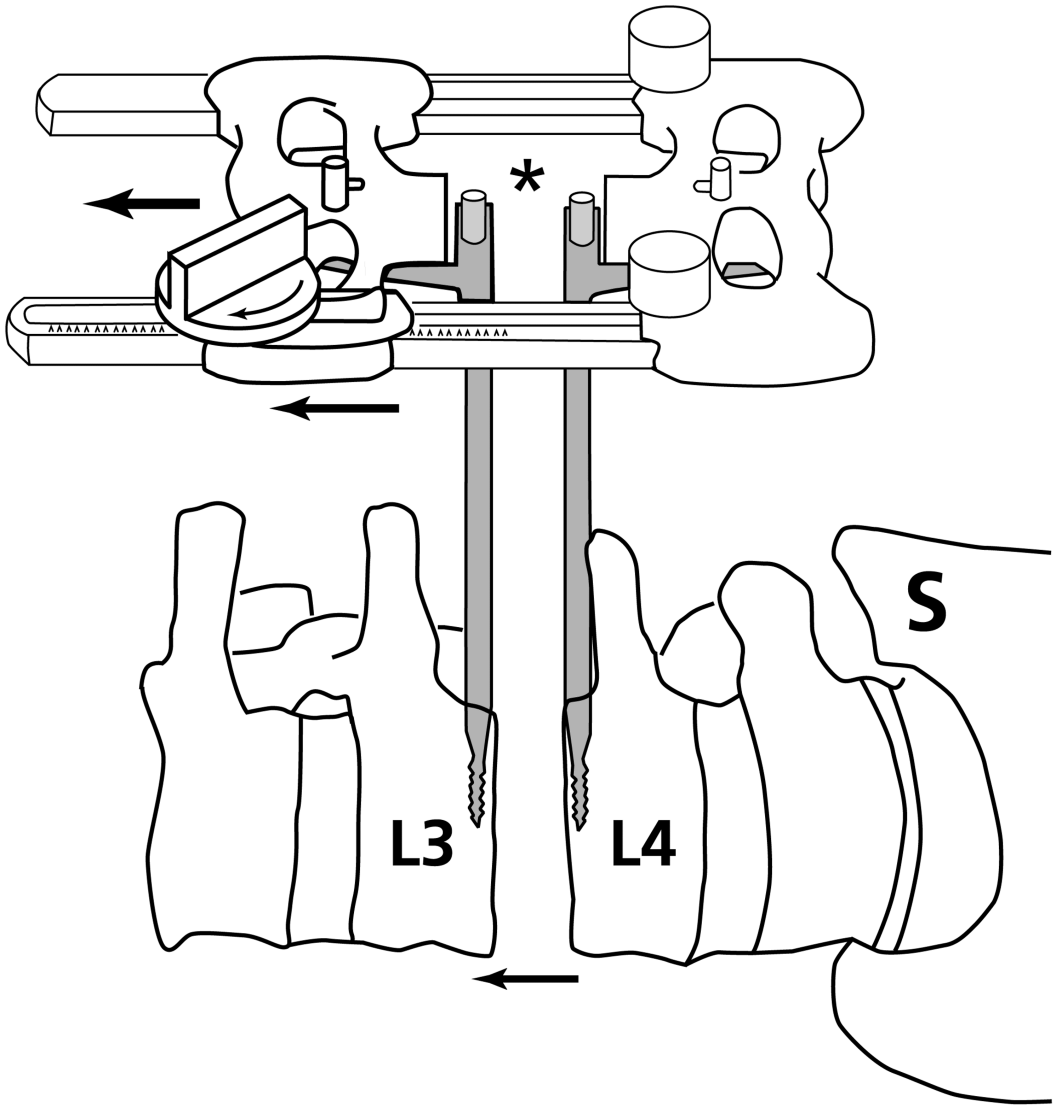
RAVINE® Lateral Access System. Illustration of RAVINE® Lateral Access System (Stryker Corp., Kalamazoo, MI, USA). In this illustration the device is placed laterally at a mid lumbar (L) spine level (S = sacrum). After the pins have been inserted into the adjacent vertebral bodies, the device is adjusted to distract the disc space (arrows). The discectomy and fusion is performed through a space that is created by retractor blades (not shown) that are inserted in the area indicated by the asterisk. The blades are then used to spread open the psoas muscle.

The final lumbar spine surgery was a two-level (L3-L4, L4-L5) discectomy and interbody fusion with posterior instrumentation and fusion (Figure 1). This procedure was performed using the "RAVINE® Lateral Access System" (Stryker Corp., Kalamazoo, MI, USA) (Figure 2). The patient was positioned in the left lateral decubitus position for a right flank transpsoas surgical approach (general aspects of this procedure can be viewed at https://www.youtube.com/watch?v=VUVfUVu4azs; accessed May 6, 2024). All surgical steps were carried out in accordance with the manufacturer’s instructions (https://youtu.be/VUVfUVu4azs; accessed May 6, 2024). Confirmation of the disc spaces was achieved with fluoroscopy. At each intervertebral level, a guide wire was placed into the disc, and dilators were placed to sweep the psoas muscle off the disc space. The RAVINE® lateral retractor was lowered so that the retractor blades and disc were parallel before the retractor blades were opened. An anterior accessory blade was placed to facilitate retraction while protecting anterior structures. A box annulotomy was then performed, and subsequently, a Cobb elevator was used to delaminate the endplates of the disc space. The far (left) side of the annulus was disrupted with an intentional mallet strike on the Cobb elevator. Disc material that still remained was removed with a large curette, ring curette, pituitary rongeur, and rasp. The same surgical steps were accomplished at both of the intervertebral levels. Interbody spacer implants (Cascadia®, Stryker Corp., Kalamazoo, MI, USA) filled with Allomatrix graft material were then impacted into the L4-L5 and L3-L4 levels, as confirmed by fluoroscopy.

Third spine surgery (posterior fusion and instrumentation) 

Through a posterior incision, the previously placed left-sided L5-S1 pedicle screws were removed, and the short rods were removed from both sides. The previous L5-S1 fusion was found to be solidly united. New pedicle screws were placed where the prior screws had been. Also, new L3 and L4 pedicle screws were placed on the left side; a Jamshidi needle was used to place guide wires prior to drilling holes for these new pedicle screws [15]. Fluoroscopy was used to confirm the appropriate placement of all implants. The L3-L4 and L4-L5 facet joints were then decorticated with a bur, and the bone shavings were used as bone graft for the posterior fusion. The entire procedure was done in 90 minutes, and the blood loss was approximately 50 mL. There were no reported perioperative complications, and the surgeon denied any plunging or untoward placement of surgical instruments that may have caused direct or indirect injury to the pancreas, which has been reported previously (discussed below) [16,17].

**Figure 3:**
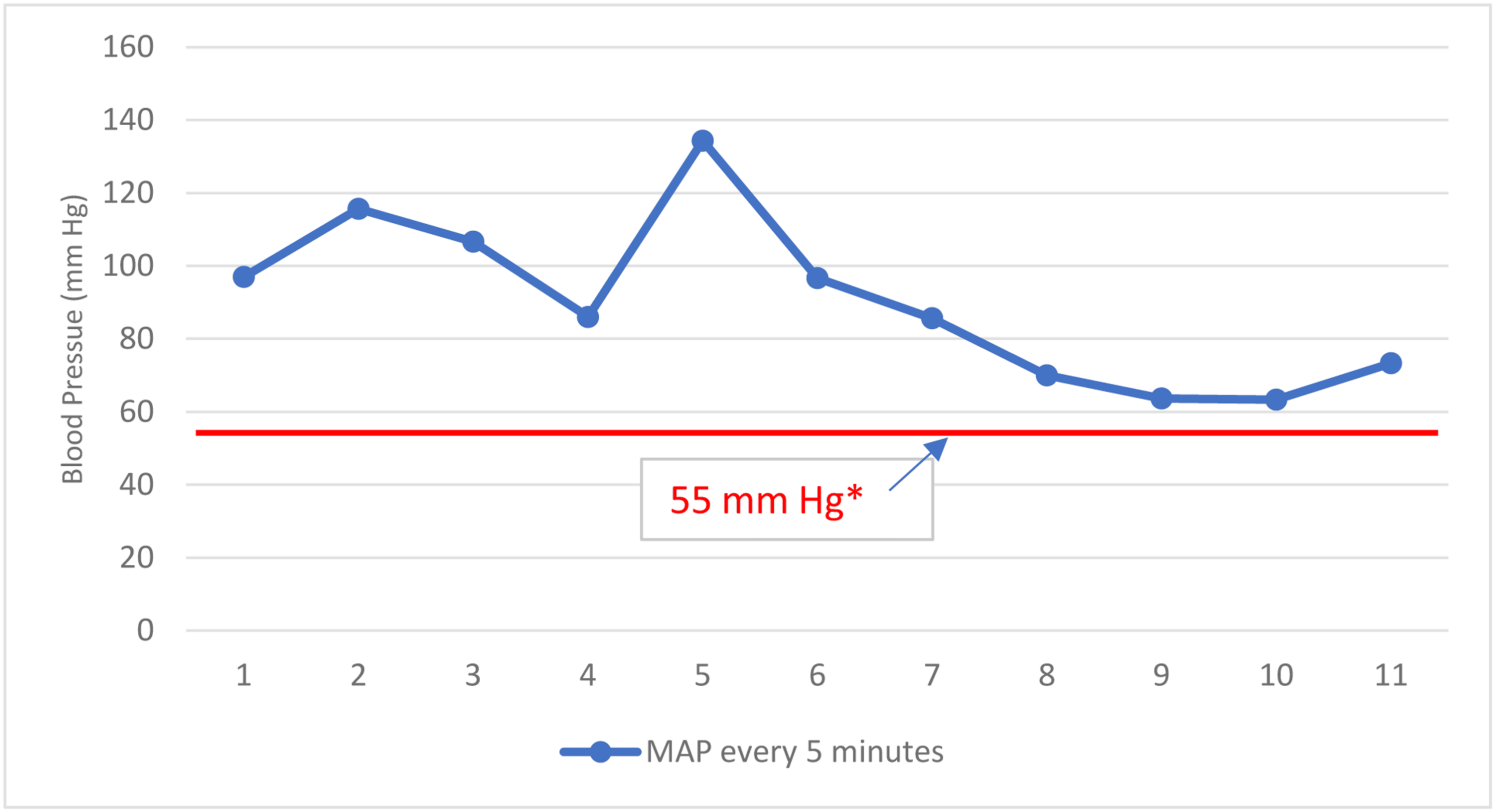
MAP during our patient's surgery. Low MAP during our patient’s spinal surgery is seen as the final four measurements (dots) in this figure. *55 mmHg is the proven threshold for significant risk of acute kidney injury during surgery, as discussed in the text. MAP = (systolic pressure + 2•diastolic pressure)/3 MAP: mean arterial pressure.

During the preparation of this case report, we examined the anesthesia record and found that the patient experienced low mean arterial pressure (MAP) (≤73 mmHg) for 20 minutes during the procedure, with one measurement at 63 mmHg (Figure 3). These measurements were obtained using an arm blood pressure cuff. The anesthesiologist considered these relatively low MAP to be inconsequential, and the surgeon was not informed that this had occurred. The implications of these relatively low MAP are discussed below. The patient was discharged from the hospital one day after surgery without any apparent complications.

Readmission to hospital three days after third lumbar spine surgery

On postoperative day 3, the patient was readmitted to the hospital for a one-day history of progressively worsening moderate-to-severe malaise, upper abdominal pain, nausea, mild shortness of breath, and an episode of syncope. Her vital signs were within normal limits, and she was afebrile. CT scanning revealed a left pleural effusion, as well as fluid collections in the left retroperitoneum (7 x 4 x 3 cm) and in the deeper integument beneath the surgical incision. All three areas were aspirated, and the results are shown in Table 1. Notably, the pleural and retroperitoneal fluids showed elevated white blood cell counts, but cultures did not show any growth.

**Table 1:** Aspirations taken upon readmission to the hospital (three days after lumbar spine surgery).

Aspiration location	Volume	Appearance	WBC	Amylase	Lipase
Pleural fluid	500 mL	Dark-brownish clear	5,740/mcL (normal range <1,000/mcL)	-	-
Retroperitoneal fluid	50 mL	Dark gray, foul-smelling	2,255/mcL (normal range <500/mcL)	4,524 units/L (normal is much less than serum normal range of 25-85 units/L)	59,034 units/L (normal is much less than serum normal range of 10-70 units/L)
Blood in the deep integument beneath the surgical incision site	60 mL	Dark	<1,000/mcL	-	-

Abnormal and other notable laboratory values from this readmission are shown in Table 2. Additionally, she was negative for 1,3-β-D-glucan, which, when present, suggests invasive fungal infection. Intravenous piperacillin/tazobactam was given for presumed bacterial infection.

**Table 2:** Notable laboratory values taken upon readmission to the hospital (three days after lumbar spine surgery*). CRP: C-reactive protein; ESR: erythrocyte sedimentation rate; BUN: blood urea nitrogen; GFR: glomerular filtration rate. *Unless indicated otherwise, all lab results listed were obtained three days after lumbar spine surgery.

Test	Result	Normal range
WBC	23.7 K/mcL	3.6-10.6
CRP	31.0 mg/dL	0-1.5
ESR	85 mm/h	Normal: <31
Procalcitonin	0.34 ng/mL	0.0-0.09
Sodium	127 mmol/L	137-146
Potassium	3.4 mmol/L	3.5-5.2
Chloride	97 mmol/L	102-111
CO_2_	16 mm/L	19-30
Glucose	115 mg/dL	65-99
BUN	32 mg/dL	8-20
Creatinine	1.42 mg/dL	0.60-1.10; 30 minutes later was 1.50 mg/dL and normalized to 0.78 mg/dL by the next day
Creatinine GFR	37 mL/min/1.73 m^2 ^	Normal: >60
Serum lipase	79 units/L	10-70; measured seven days after surgery
Serum amylase	70 units/L	25-85; measured seven and nine days after surgery

Elevated serum lipase levels were noted seven days after surgery (Table 2). Serum amylase was measured on the seventh and ninth day after surgery and was found to be normal. Additionally, amylase and lipase levels in the retroperitoneal fluid were found to be elevated (Table 1).

**Figure 4:**
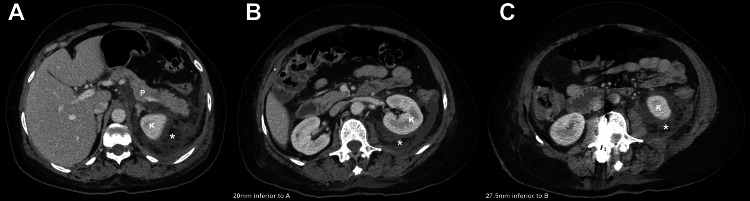
Axial CT images of our patient's abdomen. (A) The level near the T12-L1 disc space, and the tail of the pancreas can be seen. (B) The L2 level and is 20 mm inferior (i.e., closer to the sacrum) when compared to (A); the pancreas is not seen. Hence, her pancreas is mostly anterior to the L1 vertebral body. (C) The level of the L3 vertebral body and is 27.5 mm inferior to (B). Axial CT images were obtained six days after our patient’s lumbar spine surgery (November 20, 2022). Retroperitoneal fluid, indicated by the asterisk, can be seen near the left kidney. P: pancreas; K: kidney.

After three days of continued abdominal distension, pain, and rising WBC, the patient was transferred to a tertiary hospital for advanced care. Prior to the transfer, the medical team considered an acute postoperative infection to be the most likely cause of her symptoms, which is not surprising because conventional diagnostic criteria for establishing the diagnosis of pancreatitis had not been met [18]. At the tertiary care center, six days after her lumbar spine surgery, magnetic resonance imaging (MRI) of the lumbar spine showed left perinephric soft tissue swelling. CT of the abdomen and pelvis showed retroperitoneal fluid surrounding the pancreas measuring 5.7 x 1.3 cm in one image and 9.0 x 2.0 cm in another (Figure 4). MR cholangiopancreatography (MRCP) demonstrated a patent pancreatic duct. Because an acute postoperative infection was still considered a very likely possibility, piperacillin/tazobactam was discontinued, and the antibiotic regimen was broadened to include IV vancomycin, cefepime, and Flagyl. The following day, a chest tube was placed to drain the left-sided pleural effusion. A percutaneous drain was also placed to drain the retroperitoneal fluid.

On the eleventh day after lumbar spine surgery, a one-time dose of octreotide (a somatostatin analog) was given to assuage symptoms by reducing pancreato-biliary secretion from a suspected pancreatic duct leak or related injury [1,19-21]. Fourteen days after the lumbar spine surgery, the chest tube was removed, and she was discharged from the hospital to her home with the retroperitoneal drain in place. Over the next few weeks, the patient continued to have mild nausea and a poor appetite. She followed up with a primary care physician and a gastrointestinal specialist (RCM). At the patient’s three-week postoperative follow-up, a chest radiograph showed resolution of the pleural effusion. The next day, one month after her spine surgery, abdominal and pelvic CT scans with IV contrast demonstrated persistent retroperitoneal fluid with evidence of inflammation in the posterior pancreas. The spinal hardware was observed to be intact. CT scans taken 15 and 25 days later showed enlargement of the fluid collection, which was then considered to be a pancreatic pseudocyst.

**Figure 5:**
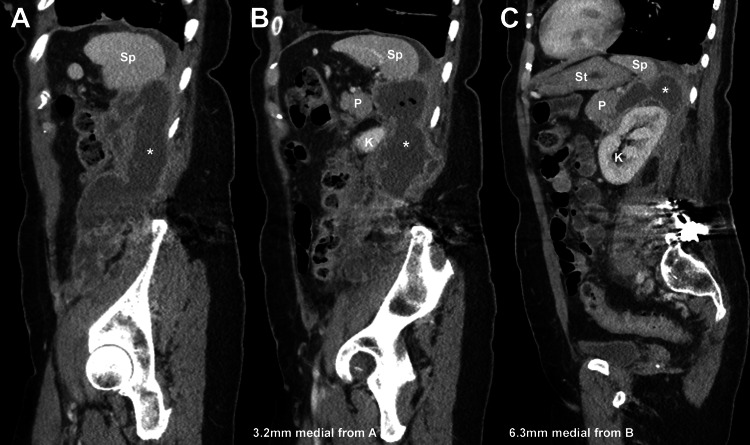
Sagittal CT images of our patient's abdomen. (A) The plane that includes the medial aspect of the left femoral head. (B) The medial aspect of the hip joint and is 3.2 mm medial from (A). (C) The sacrum is now seen because the image is 6.3 mm medial from (B). Sagittal CT images were obtained three months after our patient’s lumbar spine surgery (February 14, 2023). The pancreatic pseudocyst is indicated by the asterisk. P: pancreas; K: kidney; Sp: spleen; St: stomach.

On January 9, 2023, nearly two months after lumbar spine surgery, the retroperitoneal drain was exchanged for a larger diameter drain in order to enhance fluid egress. At that time, the gastrointestinal surgeon (RCM) recommended internal drainage of the pseudocyst (i.e., an open Roux-en-Y cystojejunostomy). On January 12, the patient sought a second opinion from another gastrointestinal specialist at a nearby academic medical center. The recommendation was to monitor drain output for another one to three months and then internal drainage should be done if there was no resolution. An abdominal CT angiogram study performed on February 14, 2023, showed enlargement of the pancreatic pseudocyst, which now extended from the pancreas and left retroperitoneal area to the iliac fossa and measured nearly 19 cm in length (Figure 5). The pancreas exhibited changes consistent with inflammation without necrosis, and there was no dilatation of the pancreatic duct. At that time, the patient complained of achy abdominal pain with mild bulging along the left abdomen.

**Figure 6:**
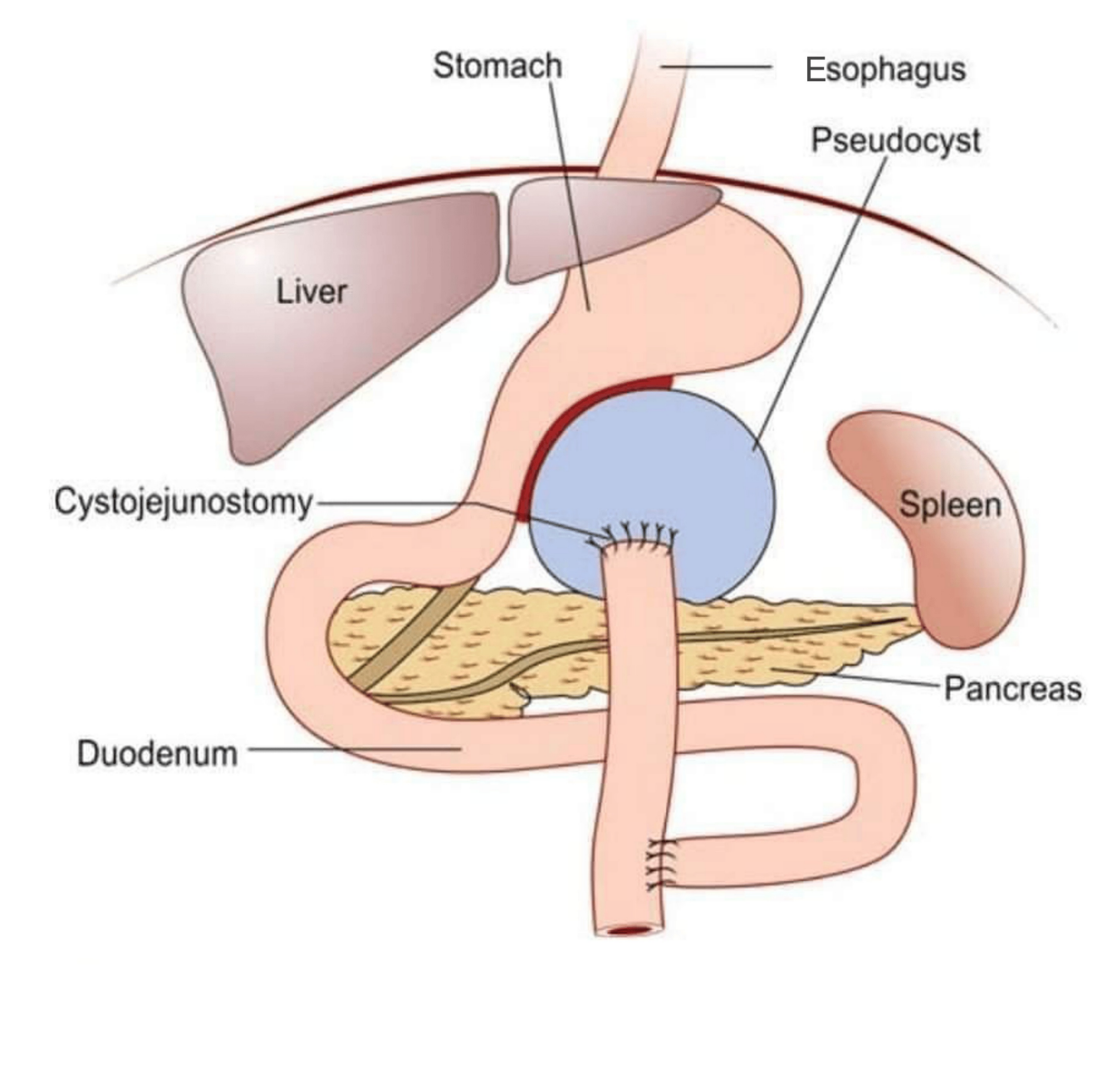
Diagram of cystojejunostomy. Diagrammatic depiction of the re-routing of jejunum for a Roux-en-Y pancreatic cystojejunostomy. Used with permission in accordance with open access under the terms of the Creative Commons Attribution License; SRB’s Manual of Surgery, Fourth Edition [22].

On March 20, 2023, four months after lumbar spinal surgery, the patient underwent an open Roux-en-Y pancreatic cystojejunostomy (which was performed by RCM) (Figure 6). During this procedure, a sample of pancreatic tissue was biopsied for histopathological examination. The pathology report described the pancreatic tissue as “soft, diffluent, and yellow,” and the microscopic description was “necroinflammatory debris.” The patient was discharged from the hospital on the fourth postoperative day.

Recovery was uneventful until postoperative day 18, when she began experiencing fevers, chills, and worsening left-sided flank and back pain. She presented to the emergency department with these symptoms on April 10, 2023, 21 days after the cystojejunostomy. She had leukocytosis (15.6 K/mcL), hypokalemia (2.9 mEq/L), mild anemia (hemoglobin 9.1 g/dL, hematocrit 28%), and low albumin (3.4 g/dL; normal range: 3.5-5.2). A CT scan showed a probable abscess extending from the retroperitoneal soft tissues. She was admitted to the hospital for percutaneous drain placement and discharged the next day with a five-day course of oral amoxicillin-clavulanate. The culture from this abscess grew *Prevotella* species, *Streptococcus constellatus*, *Eikenella corrodens*, and *Streptococcus anginosus*. Eight days later, the abscess drain was removed as there was little daily fluid collection. The next day the patient had her 30-day postoperative clinic follow-up from the cystojejunostomy surgery. Although she was generally improving, she continued to experience nausea, which was managed with oral ondansetron and omeprazole. The nausea became less frequent and generally manageable. By May 2023, the patient had improved sufficiently to tolerate out-of-state travel, and by June 2023, she was tolerating a regular oral diet.

In March 2024 (12 months after the cystojejunostomy), the patient reported that despite taking daily ondansetron and omeprazole for nausea, she had been experiencing frequent dull epigastric pain with nausea and episodes of changes in bowel habits, including constipation and diarrhea. She consulted with a gastroenterologist who performed an esophagogastroduodenoscopy (EGD) that revealed mild gastritis secondary to gastric reflux. A swallowing study showed normal gastric emptying [23]. Sucralfate was prescribed for nausea symptoms but was not helpful; mirtazipine was then used successfully [24].

At final follow-up in May 2024, she was again tolerating a regular diet and reported her overall health as “fair.” She completed the Short Form 36 Health Survey (SF-36) [25], which was compared to one completed six months before her final lumbar spine surgery. The results of the SF-36 are shown in Table 3. Overall, her 2024 scores decreased in each of the eight categories evaluated by the SF-36 (note that increased scores are considered favorable).

**Table 3:** SF-36 results*. SF-36: Short Form 36 Health Survey. *All items are scored so that a high score defines a more favorable health state. In addition, each item is scored on a 0-100 range, with the lowest and highest possible scores are 0 and 100, respectively. Aggregate scores are compiled as a percentage of the total points using the RAND scoring table.

Scale	Results six months prior to last lumbar spine surgery	Results 18 months after last lumbar spine surgery
Physical functioning	80	75
Role limitations due to physical health	50	0
Role limitations due to emotional problems	100	33.3
Energy/fatigue	80	55
Emotional well-being	68	64
Social functioning	87.5	62.5
Pain	45	55
General health	61	36

## Discussion

When postoperative pancreatitis occurs in adults, it is typically after surgeries involving the pancreas or areas surrounding the pancreas. In these cases, pancreatitis can range from minor and transient to a more serious or life-threatening complication [26-29]. There are reported cases in adults where pancreatitis also developed after surgeries remote from the pancreas, for which the incidence is also very low. For example, Liu et al. [30] looked at 170 adult patients undergoing total knee arthroplasty and found that 4.7% of these patients had postoperative "biochemical pancreatitis" (defined as having serum amylase five times above the high normal level). Only one of those patients was diagnosed with "clinical pancreatitis," where clinical symptoms of pancreatitis were also present.

As noted in the Introduction section, in adults, the occurrence of acute pancreatitis soon after spinal surgery is rare, with only a few reports of this condition as a complication of corrective scoliosis surgery [1,7,8] and even fewer cases acutely after non-scoliosis thoracolumbar or lumbosacral spinal surgery [16,17,31-33] (Table 4). In all adult cases that we located where pancreatitis occurred as a complication of spine surgery, the pancreatitis was acute; in one case, there was pancreatic necrosis from hypotension. As noted, in one of these cases, the pancreatitis became recurrent and eventually resolved [8]. To our knowledge, there are no reported cases of chronic or recurrent pancreatitis following non-scoliosis thoracolumbar, lumbar, or lumbosacral surgery in adults that also resulted in a pancreatic pseudocyst requiring external or internal drainage. In this context, our patient’s case is unique. Below we discuss various hypothetical factors that might have individually, or in combination, contributed to the emergence of our patient’s postoperative pancreatitis. After discussing those various factors, we then focus our discussion on the emergence and management of her pancreatic pseudocyst.

**Table 4:** Selected cases in adults who developed acute postoperative pancreatitis after spine surgery. *Additional cases published in Japanese can be found in Tauchi et al. [31] and general mention of young adults can be found in Feng et al. [1]. NA: data not available.

Reference	Patient age, sex	Scoliosis?	Type of surgery	Indication for surgery	Complication post-surgery	Potential cause	Post-surgery intervention	Outcome
Korovessis et al. [8]	28, Female	Yes	Anterior fusion and instrumentation L2-L4	Polioscoliosis	Recurrent pancreatitis (post-surgery six, 16, and 32 months)	-	Conservative treatment	Full recovery within weeks
Rajaraman et al. [17]	60, Female	No	Anterior lumbar interbody fusion and instrumentation L4-L5	Spondylolisthesis	Acute pancreatitis	Suboptimal placement of self-retaining retractor; presumed direct trauma	Conservative treatment	Full recovery within 10 days
Tauchi et al.* [31]	53, Female	No	Posterior interbody fusion and instrumentation L3-L5	Spondylolisthesis	Acute pancreatitis	Pancreatic ischemia due to intraoperative blood loss	Conservative treatment	Full recovery within weeks
Kobayashi et al. [7]	53, Female	No	Posterior interbody fusion and instrumentationL3-L5	Spondylolisthesis	Acute pancreatitis	NA	NA	NA
Rathod et al. [16]	65, Female	Yes	Posterolateral decompression and instrumentation T9-T10 and L1-L2	T10-11 vertebral body destruction secondary to tuberculosis	Acute pancreatitis secondary to pancreatic laceration	Cobb elevator plunge of 15 cm near L1-2 transverse processes	Exploratory laparotomy and pancreatic laceration repair	Full recovery within weeks

Gallstone theory

Leading causes of pancreatitis include gallstones and alcohol use disorder [11,12,14,34]. Although our patient did not have these conditions in proximity to any of her lumbar spine surgeries, it is important to consider the rare possibility that choledocholithiasis was in play, even though she had a remote cholecystectomy. In patients with an intact gallbladder, the most common cause of acute pancreatitis is that a gallstone exits the cystic duct and lodges at the pancreatobiliary junction or at the ampulla of Vater. This blockage then causes inflammation and/or bile reflux into the pancreatic duct, resulting in pancreatitis [35]. In most cases when choledocholithiasis occurs after cholecystectomy, the stones are residual and missed during surgery, as opposed to new stone formations [36]. In reported cases where residual stones were found, the majority were identified within three years of the cholecystectomy [37]. Some reports of choledocholithiasis more than 10 years after cholecystectomy describe stones being present within the remnant of the cystic duct, or stones that formed secondary to migration of surgical clips that became a nidus for stone formation [36,38]. There are other studies that revealed stones of the common bile duct in association with bile duct stricture, periampullary diverticulum, parasites or foreign bodies within the bile duct, or other factors that can cause bile stasis [37]. There are also notable, though rare, cases of choledocholithiasis occurring many years after cholecystectomy, including, among others: (1) 15 years later in a 86-year-old male [39], (2) 33 years later in a 57-year-old female [40], and (3) 40 years later in a 72-year-old female [41]. However, the rare possibility that our patient might have developed gallstone pancreatitis and passed the gallstone in the days following her spinal surgery is a nearly impossible explanation for her long-term pancreatic symptoms, as gallstones are not known to cause chronic pancreatitis [42,43]; additionally, CT scanning in our patient did not reveal evidence of gallstones.

Intraoperative ischemia theory

Another possible cause of our patient’s pancreatitis is ischemic damage to the pancreas due to intraoperative hypoperfusion from acute blood loss. In a study of postoperative pancreatitis, White et al. [44] reported on 70 adult patients (mean age unknown) who were diagnosed with acute pancreatitis, representing 9.5% of all pancreatitis cases diagnosed in their surgical unit over the same time period. Seventeen of these patients had undergone procedures at sites remote from the pancreas, including lower abdominal surgery in 10, transurethral prostatic resections in three, and mastectomy, parathyroidectomy, hip prosthesis, and laminectomy in one patient each. While they did not identify a definitive etiology of postoperative pancreatitis in these cases, they hypothesized that hypovolemic shock possibly causing the formation of microthrombi resulting in decreased blood flow to the pancreas may be a cause of postoperative pancreatitis after surgery in a majority of their patients. This suggestion is supported by a study of pancreatitis after scoliosis correction surgery in children and young adults (six of 44 patients; 14%), showing that intraoperative blood loss was much higher in pediatric and adult patients who had high amylase and/or developed pancreatitis [3]. However, these findings are controversial considering Borkhuu et al.’s [4] study of 355 pediatric patients (average age: 14) who had posterior and anteroposterior fusions for scoliosis. This study found no difference in intraoperative blood loss between the patients with (n = 109) and without (n = 246) postoperative pancreatitis (p = 0.47).

Another source of hypoperfusion to the pancreas may be a result of low MAP intraoperatively. Feng et al. [1] identified low MAP as an independent risk factor of postoperative pancreatitis. They reported on 176 patients (122 female and 54 male) with a mean age of 17 years (range: 3-58 years) who had scoliosis correction. They calculated MAP as follows: (systolic pressure + 2•diastolic pressure)/3. The MAP in the group with pancreatitis was 57.9±6.5 and without pancreatitis was 66.1±7.8 (p < 0.001). Additional evidence supporting this hypoperfusion theory can be found in studies of acute pancreatitis secondary to ischemia from various causes of surgery-related hemorrhage (these cases excluded spine and pancreas-related surgeries) [45,46]. 

While it is unlikely that our patient experienced ischemic damage to the pancreas secondary to intraoperative blood loss (her blood loss was only 50 mL), she may have experienced ischemic damage secondary to low MAP. Our patient’s mean MAP remained greater than 65 mmHg for 80 of the 90-minute duration of her surgery, which is the generally accepted definition for intraoperative hypotension in non-cardiac surgical cases [47]. The mean MAP for her entire case was 90 mmHg (range: 63-134 mmHg, median: 86 mmHg). However, as shown in Figure 3, she experienced MAP as low as 63 mmHg intraoperatively and remained at MAP ≤73 mmHg for 20 minutes. The possibility that these lower MAP could have had a deleterious effect on her pancreas can be inferred from perioperative data for 33,330 non-cardiac surgeries at the Cleveland Clinic, Ohio, USA [48]. In that comprehensive study, Walsh and colleagues [48] reported data from non-cardiac surgeries (types of non-cardiac surgeries were not specified) showing that 55 mmHg is the threshold for significant acute kidney injury (AKI). Although they did not study acute pancreatic injury, there is evidence that the pancreas is more sensitive to ischemia than the kidney [49]. They evaluated the association between postoperative AKI and intraoperative MAP from less than 55, and between 55 and 75 mmHg, to determine the threshold of MAP where the risk of AKI is increased. AKI occurred after 2,477 surgeries (7.4%), of which 2,043 (82.4%) occurred within three days of surgery. The results showed that compared with patients who never developed a MAP less than 55 mmHg, patients with a MAP less than 55 mmHg for 1-5, 6-10, and 11-20 minutes had graded increases in risk (1.18 [95% CI, 1.06-1.31], 1.19 [1.03-1.39], and 1.32 [1.11-1.56]), respectively. As noted, patients who experienced intraoperative MAP less than 55 mmHg for even short durations (1-5 minutes) during non-cardiac surgery had an increased risk for developing AKI compared to those whose MAPs were higher. While MAPs of 60-69 mmHg did not show a statistically significant difference in manifesting ischemic kidney injury compared to baseline, the authors did not provide exact p-values so that statistical trends could be identified if present. Nevertheless, Walsh et al. showed increased risk of AKI when MAP was 60-90 mmHg, and the risk increased with the amount of time spent within range. In view of their results, it can be estimated that when MAP fell below 70 mmHg, there was ~5% chance (~1 in 20) of developing AKI post-surgery.

At three days after spinal surgery, our patient had elevated creatinine levels (1.5 mg/dL, normal range: 0.6-1.1 for women). Serum creatinine measurements are the standard for the diagnosis of AKI, with creatinine levels of 1.2 or higher providing reason for concern of AKI in women [50]. Furthermore, ischemia is the most common cause of AKI [51]. While studies noted in the foregoing discussion do not comment directly on the relationship between MAP and pancreatic ischemia, it is reasonable to suspect that our patient’s pancreas may have experienced ischemic damage during the same period that her kidney experienced significant ischemia. Clinical data suggest that intraoperative ischemia to the pancreas can result in pancreatitis within one to three days of surgery [1,48]. For example, Walsh et al. reported that 82.4% of their cases of AKI occurred within three days of surgery [48].

Nevertheless, ischemic pancreatitis is rare and has even been suggested as a diagnosis of exclusion [45,46]. Case reports describing ischemic pancreatitis typically note that this results from events with large blood loss or diversion, such as abdominal aortic aneurysm, hemorrhage, and mesenteric vein occlusion [45,52]. From these perspectives, ischemic damage to the pancreas without massive blood loss or other mechanisms of occlusion seems to be an unlikely, though not impossible, explanation for the cause of our patient’s postoperative pancreatitis.

Intraoperative trauma theory

Another uncommon, but plausible, cause of postoperative pancreatitis in our patient’s case is direct trauma to the pancreas intraoperatively. In surgeries that are done in the vicinity of the pancreas, such as intra-abdominal surgeries, there are reported cases where direct surgical trauma appeared to cause postoperative pancreatitis [34]. There are also rare cases in adults where traumatic pancreatitis resulted from surgeries seemingly remote from the pancreas, such as lumbar spine surgery [16,17]. For example, a case report by Rajaraman et al. [17] describes the suboptimal placement of a self-retaining retractor during L4-L5 spinal fusion surgery in a 60-year-old female patient that they suggested may have caused direct traumatic injury to the pancreas resulting in acute pancreatitis. The patient underwent an anterior lumbar interbody fusion at the L4-L5 level through a left lateral retroperitoneal approach as described previously. The exposure of the interspace and discectomy was uneventful, blood loss was 300 mL, and the patient was at all times hemodynamically stable. Although the surgery was uneventful, the patient developed nausea, vomiting, and abdominal distension three days later and was found to have serum amylase elevated to four times the normal range and serum lipase elevated to six times the normal range. A CT scan showed swelling at the tail of the pancreas and a small collection of free fluid in the pelvis. Although the surgical team did not identify an exact cause of pancreatitis, they felt that in view of lacking another explanation suboptimal placement of the self-retaining retractor may have been the proximate cause. The patient recovered by the third postoperative week without need for surgical intervention, and there was no recurrence at the last reported follow-up one year later.

Rathod et al. [16] reported the case of a 65-year-old female patient who was diagnosed with a pancreatic laceration following posterolateral decompression and instrumentation of T9, T10, L1, and L2 levels, which was done for T10-11 tuberculosis of the spine. During surgery, a Cobb elevator slipped anteriorly around the transverse process on the left side and plunged about 15 cm. Although the Cobb elevator was removed immediately, the patient developed leukocytosis and significantly elevated serum lipase, suggestive of pancreatic injury. The patient underwent exploratory laparotomy, which confirmed the CT finding of pancreatic laceration. The laceration was repaired with non-absorbable polypropylene sutures. The patient recovered fully within weeks.

Although similar direct trauma would explain our patient’s postoperative complication, this etiology was denied by her surgeon. Additionally, our extensive search of anatomical literature confirmed that the body of the pancreas lies in the same transverse plane as the L2 vertebral body, and the pancreatic head would be the portion of the pancreas that would be closest to the level of the L2-L3 intervertebral disc while the tail is more proximal (i.e., above the L2 level) [53-57]. Thus, the pancreatic head, though closest to the region of our patient’s surgical field, is still relatively distant to her upper-most L3-L4 fusion level. Notably, evaluation of our patient’s abdominal CT scans also shows that the lower edge of her pancreas was at the level of the lower portion of the L2 vertebral body. To strike the head of the pancreas with a drill bit, retractor, or other surgical instrument, our patient’s surgeon would have had to overpenetrate by approximately 8 cm from the anterior surface of the L3 vertebral body at an upward tilt of about 30°. These facts support the improbability of this as a cause of her pancreatitis.

Low BMI theory

In a study of adolescent patients who had surgery for idiopathic scoliosis, Laplaza et al. [2] found that postoperative pancreatitis was more common in patients with low BMI (19 vs. 23) (p = 0.04). Feng et al. [1] studied 176 patients between the ages of three and 58 years old who had scoliosis correction and found that patients with postoperative pancreatitis had a lower BMI than patients without pancreatitis (15.5 vs. 19.5, p < 0.001). There was no significant difference in age between patients who did and did not develop acute postoperative pancreatitis. It has been suggested that blunt pancreatic injury is more common in children and young adults because they have a thinner or absent mantle of protective fat surrounding the pancreas compared to older adults [58]. This helps explain why there is an increased risk of acute pancreatitis in patients with significantly lower BMI who have surgery to correct scoliosis deformity. However, it seems unlikely that these mechanisms were at play in our patient because (1) she always had a normal BMI (24.6; normal range: 18.5-24.9) [59] and (2) manipulations that would be needed when there are deformities (e.g., scoliosis or significant spondylolisthesis) were not needed during her spine surgery.

Drug-induced pancreatitis theory

In our patient’s case, it is also important to consider drug-induced pancreatitis because this accounts for 0.1-5% of cases of acute pancreatitis, as shown in studies of these cases in the general population (i.e., not with respect to postoperative cases) [60,61]. The drugs implicated in causing pancreatitis include angiotensin-converting enzyme (ACE) inhibitors, statins, oral contraceptives/hormone replacement therapies (HRTs), diuretics, highly active antiretroviral therapy, valproic acid, and hypoglycemic agents [61]. Our patient was taking HRT (estradiol) at the time of her surgery, which might trigger pancreatitis by causing hypertriglyceridemia [62]. Still, this is a very rare cause of pancreatitis, with few cases reported worldwide and few case-control studies exploring the association between pancreatitis and HRT [62,63]. In a prospective study involving 31,494 postmenopausal women (aged 48-83 years) from the population-based Swedish Mammography Cohort, Oskarsson et al. [63] found an increased risk of pancreatitis with the use of post-menopausal HRT. In contrast, Tetsche et al. [64] found only a slightly elevated risk of pancreatitis in patients with past (not current) combined estrogen/progesterone HRT use and no increased risk of pancreatitis in patients taking estrogen-only HRT. Additionally, drug-induced pancreatitis is usually defined if the patient meets the following criteria: (1) acute pancreatitis occurs during the administration of a drug, (2) symptoms of acute pancreatitis disappear after drug withdrawal, (3) symptoms recur after a rechallenge of the suspected drug, and (4) all other common causes are excluded [60]. Our patient had been taking estradiol for several years, making it unlikely that the drug had suddenly precipitated her pancreatitis.

Development and management of pancreatic pseudocysts

Although it is unusual that our patient developed chronic pancreatitis following her non-scoliosis spinal surgery, it is not unusual that she developed a pancreatic pseudocyst as a result of her chronic pancreatitis. Pseudocysts occur as a complication of acute pancreatitis in 5-15% of patients and as a complication of chronic pancreatitis in 20-40% of patients [65]. A pseudocyst differs from a true cyst in that it is a fluid collection that lacks an epithelial lining and instead is lined by fibrous and granulation tissues [66]. Although pancreatic pseudocysts usually self-resolve or remain asymptomatic, there is risk for rupture or infection in larger cysts and in those that are non-responsive to external drainage [10]. The first-line treatment for a pancreatic pseudocyst is percutaneous drainage, which is successful in 60-90% of cases [29]. However, in cases in which the pseudocyst is not resolved by percutaneous drainage (as in our patient), surgical intervention may be indicated [29,67]. Permanent resolution of the pseudocyst is seen in 91-97% of cases treated with internal drainage [29]. In our patient, a Roux-en-Y cystojejunostomy was considered to be the best choice for internal drainage because this procedure results in a lower incidence of recurrent pancreatitis and exocrine insufficiency as compared to other internal drainage procedures for pseudocysts [65,68]. 

Delayed diagnosis of pancreatitis

Our patient’s pancreatitis was diagnosed rather late in her postoperative course. For nearly the first two weeks after her lumbar spinal surgery, the consistent top diagnosis for her symptoms was “sepsis of unclear source.” This was thought to be the explanation for her retroperitoneal fluid collection, or “abscess” (as stated in the clinical record), as well as her leukocytosis. While leukocytosis is a classic hallmark of infection, it is also classically seen in pancreatitis [69]. Thus, it is important that when a patient presents acutely with leukocytosis and malaise, upper abdominal pain, and nausea and vomiting after spine surgery, serum amylase should be obtained, and pancreatitis should be considered prominently in the differential diagnosis.

## Conclusions

The 63-year-old female reported herein developed a very rare postoperative complication of chronic pancreatitis after a commonly performed elective lumbar spine surgery for non-scoliosis arthritis. Her chronic pancreatitis thereafter became associated with a large pancreatic pseudocyst, which was ultimately treated successfully with an open Roux-en-Y cystojejunostomy. Although there are several possible etiologies of pancreatitis occurring soon after spinal surgery in adults, we could not identify, with high certainty, the cause of our patient’s postoperative complication. Leukocytosis was detected within a few days of symptom onset; however, this was not considered as being possibly associated with pancreatitis. This was despite her chief complaints of abdominal pain, nausea, vomiting, and general discomfort, as well as high amylase levels found in her retroperitoneal fluid. Because pancreatitis can be an elusive complication of spinal surgery in an adult, healthcare providers should consider this diagnosis prominently in the differential diagnosis for the underlying cause of these early postoperative symptoms and leukocytosis. We report this case not only because of its rarity but also to alert surgeons and other healthcare providers that pancreatitis can occur acutely in the postoperative period in adults and evolve into a chronic problem (pseudocyst formation) requiring surgical correction.
